# Heat shock protein 70 selectively mediates the degradation of cytosolic PrPs and restores the cytosolic PrP-induced cytotoxicity via a molecular interaction

**DOI:** 10.1186/1743-422X-9-303

**Published:** 2012-12-06

**Authors:** Jin Zhang, Ke Wang, Yan Guo, Qi Shi, Chan Tian, Cao Chen, Chen Gao, Bao-Yun Zhang, Xiao-Ping Dong

**Affiliations:** 1 State Key Laboratory for Infectious Disease Prevention and Control, National Institute for Viral Disease Control and Prevention, Chinese Center for Disease Control and Prevention, Chang-Bai Rd 155, Beijing, 102206, People’s Republic of China; 2 Chinese Academy of Sciences Key Laboratory of Pathogenic Microbiology and Immunology, Institute of Microbiology, Chinese Academy of Sciences, Beijing, 100101, China

**Keywords:** Hsp70, Cytosolic PrP, Apoptosis, Prion disease, Geldanamycin

## Abstract

**Background:**

Although the aggregation of PrP^Sc^ is thought to be crucial for the neuropathology of prion diseases, there is evidence in cultured cells and transgenic mice that neuronal death can be triggered by the accumulation of cytosolic PrPs, leading to the hypothesis that the accumulation of PrPs in the cytosol of neurons may be a primary neurotoxic culprit. Hsp70, a molecular chaperone involved in protein folding/refolding and degradation in the cytoplasm, has a protective effect in some models of neurodegenerative diseases, e.g., Alzheimer’s and Parkinson’s diseases, but its role in prion diseases remains unclear.

**Results:**

To study the role of Hsp70 in prion diseases, we used immunoprecipitation to first identify a molecular interaction between Hsp70 and PrPs. Using immunofluorescence, we found that Hsp70 colocalized with cytosolic PrPs in HEK293 cells transiently transfected with plasmids for Cyto-PrP and PG14-PrP but not with wild-type PG5-PrP or endoplasmic reticulum (ER)-retained PrPs (3AV-PrP and ER-PrP). Using western blot analysis and apoptosis assays of cultured cells, we found that the overexpression of Hsp70 by transfection or the activation of Hsp70 by geldanamycin selectively mediated the degradation of cytosolic PrPs and restored cytosolic PrP-induced cytotoxicity. Moreover, we found that Hsp70 levels were up-regulated in cells expressing Cyto-PrP and in hamster brains infected with the scrapie agent 263K.

**Conclusion:**

These data imply that Hsp70 has central role in the metabolism of cytosolic PrPs

## Introduction

Human prion diseases include Creutzfeldt-Jakob disease (CJD), kuru, fatal familial insomnia (FFI) and Gerstmann-Straussler-Scheinker syndrome (GSS). The conversion of the normal cellular prion protein (PrP^C^) into an insoluble and protease-resistant isoform (PrP^Sc^), which propagates itself by imposing its abnormal conformation onto a PrP^C^, is fundamental to the pathogenesis of prion diseases. It has been commonly assumed that aggregation of misfolded PrP^Sc^ is the cause of neurodegeneration in prion diseases [1]. However, in several studies, significant pathology and clinical dysfunctions can develop even when there is little accumulation of PrP^Sc^. Similarly, the accumulation and a high abundance of PrP^Sc^ has been accompanied by limited symptomatology [2]. These data suggest that PrP^Sc^ may not be the major cause of neuronal dysfunction in prion diseases.

Studies of mutant PrP molecules associated with inherited prion diseases support the hypothesis that PrPs that are mislocalized to the cytoplasm acquire neurotoxic potential [3]. PG14-PrP, a PrP mutant with an extra nine-octapeptide insertion, is associated with a familial prion disease in humans, can form numerous aggregates in neutrophils, is highly neurotoxic and promotes progressive neurodegenerative disease in transgenic Tg (PG14) mice [4]. Mice expressing a PrP mutant lacking the N-terminal ER targeting signal (Cyto-PrP) acquired severe ataxia due to the rapid degeneration of cerebellar granule neurons [5]. 3AV-PrP is an artificial PrP mutant with three alanine to valine mutations in the hydrophobic domain. 3AV-PrP substantially increases the hydrophobicity, remains intracellular at either the ER or Golgi, and causes neurodegeneration in transgenic mice [6]. Expediting the removal of these mislocalized PrPs may be a relevant therapeutic strategy to reduce neurotoxicity.

Heat shock proteins (HSPs) belong to the family of chaperones and have important roles in regulating the refolding and degradation of misfolded/mislocalized proteins [7,8]. Hsp70 is the most studied member of the cytoplasmic HSP family. Recent studies in several neurodegeneration models, including Alzheimer’s disease (AD), Parkinson’s disease (PD) and Huntington’s disease (HD), suggest that the overexpression of Hsp70 can be protective. In various cellular models for AD, increasing the levels of Hsp70 inhibited the propensity of Aβ to aggregate and reduced the toxicity of Aβ. Hsp70 also promoted the solubility of tau and its binding to microtubules [9]. The aggregation of α-synuclein is a hallmark of sporadic and familial PD. The overexpression of Hsp70 prevented neuronal loss by inhibiting α-synuclein fibril formation [10]. In a yeast model for HD, the expression of Hsp70 reduced the toxicity associated with the expression of mutant huntingtin (htt) by preventing its aberrant interaction with an essential polyQ-containing transcription factor [11]. It remains unclear whether Hsp70 also plays a role in prion diseases.

Hsp70 is one of the most potent molecular chaperones. In this study, we present evidence that Hsp70 is able to directly interact with PrPs. Colocalization studies indicate that the cytosolic forms of mutant PrPs are the targets of Hsp70. Furthermore, we found that the overexpression or activation of Hsp70 in cultured cells selectively mediated the degradation of cytosolic PrPs and contributed to the protective effect against cytotoxicity induced by cytosolic PrPs. These data implicate Hsp70 as a central regulator in the metabolism of cytosolic PrPs.

## Materials and methods

### Ethics statement

The use of animal specimens in this study was approved by the Ethical Committee of the National Institute for Viral Disease Prevention and Control, China CDC under protocol 2009ZX10004-101. All Chinese golden hamsters were maintained under clean conditions. Housing and experimental protocols were performed in accordance with the Chinese Regulations for the Administration of Affairs Concerning Experimental Animals.

### Plasmids and reagents

Mammalian expression plasmids for human wild-type PrP (PG5-PrP), Cyto-PrP, ^Ctm^PrP (3AV-PrP), 9 extra octarepeats inserted PrP (PG14-PrP) and PrP-KDEL (ER-PrP) were constructed in the vector pcDNA3.1+ as described elsewhere [12]. The plasmid pEGFP-Hsp70 was obtained from Addgene (Cambridge, MA) [13]. The antibody to PrP (3F4) was from Millipore (Billerica, MA). Antibodies to β-actin (sc-47778) and Hsp70 (sc-1060) were from Santa Cruz (Santa Cruz, CA). Antibody to Calnexin (208880) was from Merck Millipore (Billerica, MA). The HRP-conjugated anti-goat and anti-mouse IgG were from Boehringer (Germany). The Alexa Fluor 568 goat anti-mouse IgG was from Invitrogen (USA). Geldanamycin (cat# 345805) and MG132 (474790) were purchased from Calbiochem (San Diego, CA).

### Transfection

Cell lines, including human embryonic kidney (HEK) 293 (CRL-1573) cells and mouse neuroblastoma Neuro-2a (CCL-131) cells, were maintained in Dulbecco’s modified Eagle’s culture medium supplemented with 10% fetal calf serum. The various recombinant plasmids were transiently transfected into HEK 293 or N2a cells using Lipofectamine 2000 (Invitrogen).

### RNA Interference

RNA interference was carried out using siRNA purchased from Invitrogen. The siRNA corresponding to the Hsp70 mRNA sequences 5’-AGG ACG AGU UUG AGC ACA ATT-3’ was used to inhibit endogenous Hsp70 protein expression. The negative control siRNA has no relation with human gene (sense siRNA3, 5’-UUC UCC GAA CGU GUC ACG UTT-3’ and antisense siRNA3, 5’-ACG UGA CAC GUU CGG AGA ATT-3’). Various siRNAs were transfected into HEK 293 cells using Lipofectamine 2000 (Invitrogen, Carlsbad, CA). Cells were collected for further experiments 72 h after transfection.

### Preparation of hamster brain homogenates and cell lysates

Hamster brain tissues were homogenized in cold lysis buffer (1% Nonidet P-40, 50 mM HEPES, pH 7.6, 10% glycerol, 1 mM EDTA, 20 mM β-glycerophosphate, 1 mM sodium orthovanadate, 1 mM sodium metabisulfite, 1 mM benzamidine hydrochloride, 10 μg/ml leupeptin, 20 μg/ml aprotinin and 1 mM phenylmethylsulfonyl fluoride), followed by sonication [14]. Cultured cells treated with different transfections or reagents were harvested, and the whole cell lysates were prepared in cold lysis buffer with sonication. After centrifugation at 10,000 × *g* at 4°C for 10 min, the supernatants were collected for further experiments.

### Western blots

Cellular lysates or brain homogenates were separated by 12% SDS-PAGE and electro-transferred onto nitrocellulose membranes. After blocking with 5% nonfat-dried milk in PBST (phosphate buffered saline, pH 7.6, containing 0.05% Tween-20), the membranes were incubated with a PrP-specific monoclonal antibody (mAb) 3F4 (Millipore), diluted 1:4000; a mAb to Hsp70, diluted 1:2000; or a mAb to human β-actin (Santa Cruz), diluted 1:2000. Subsequently, the membranes were incubated with HRP-conjugated anti-mouse IgG or anti-goat IgG, diluted 1:10,000. The reactive signals were visualized by ECL (PE Applied Biosystems, Foster City, USA). Immunoblots were quantified using a scanning densitometer in conjunction with the NIH ImageJ software. The signals were normalized to the loading controls.

### Immunoprecipitations

Immunoprecipitations (IPs) were carried out using whole cell lysates (400 μg of total protein), 2–4 μg of antibody and 20 μl of Dynabeads®-coated Protein G (Invitrogen). The cell lysates were mixed with different antibodies at 4°C for 3–4 h and subsequently incubated with Dynabeads®-coated Protein G for another 2 h. The immunocomplexes were collected by a short spin and washed five times in wash buffer before being resolved by SDS-PAGE. The complexes were detected by Western blotting.

### Apoptosis assays

The situations of the mitochondrial transmembrane transition in live cells were determined with a MitoCaptureTM Mitochondrial Apoptosis Detection kit, BioVision (Plymouth meeting, PA, USA) according to the manufacturer’s instruction. Growing cells (approximately 60% confluence) were transiently transfected with various recombinant plasmids. The cells were collected 48 h after the transfection, resuspended in 1 ml of diluted MitoCapture solution, and then incubated at 37°C for 20 min. After a short spin, the cells were resuspended in 1 ml of pre-warmed incubation buffer. The cells were mounted onto glass coverslips and examined using fluorescence microscopy (Olympus BX51, Japan). The total number of cells in a field of view were counted using ImageJ. Apoptotic and healthy cells displayed as green and red, respectively. The percentage of apoptotic cells was defined as the number of green cells vs. the total number of cells.

### Immunofluorescence staining

The cells were fixed with formaldehyde (4% paraformaldehyde, freshly depolymerized in 0.1 M sodium phosphate buffer, pH 7.4) at RT for 15 min and washed three times in PBS. The cells were blocked with blocking buffer (PBS with 5% FBS and 0.1% Triton X-100) at RT for 1 h and then incubated with a 1:200 dilution of anti-PrP mAb in PBS with 2% BSA and 0.3% Triton X-100 at 4°C overnight. The cells were then washed and incubated with a 1:200 dilution of the appropriate secondary antibody (Alexa Fluor® 568 Goat anti-Mouse, Invitrogen) at RT for 2 h. After washing, the cells were incubated with 0.5 mg/ml DAPI (Invitrogen) at RT for 2 min. The cells were sealed, and images of the targeting proteins were analyzed using confocal microscopy (Leica ST2, Germany).

### Extract subcellular proteomes

Cells were fractionated into cytosol, membranes/organelle mitochondria, nuclei, and cytoskeleton using Subcellular ProteoExtract kit from Merck Millipore according to the manufacturer's directions.

### Statistical analysis

In this study, all of the experiments were conducted at least 3 times with consistent results. The mean value and standard error of multiple data points or samples were used to represent the final result. Student’s *t* test was used for statistical analysis and a P < 0.05 was considered significant.

## Results

### The identification of cytosolic PrPs as a target for Hsp70

**Figure 1:**
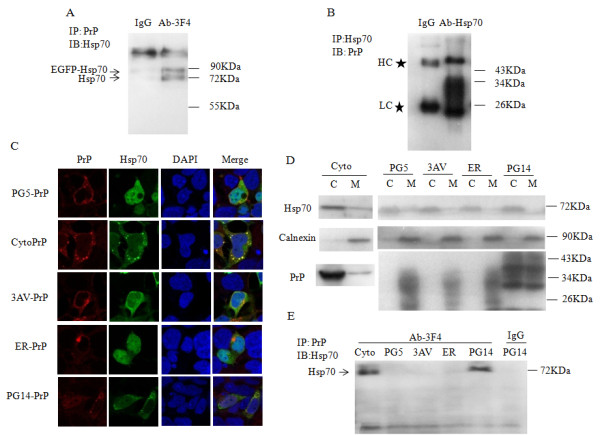
**The cytosolic forms of PrP were targets of Hsp70. A** and **B**. Immunoprecipitation assays. HEK293 cells were co-transfected with pcDNA-PrP-PG5 and pEGFP-Hsp70. Forty-eight hours after the transfection, the cells were harvested, and 400 μg of the whole cell lysate proteins was used in the immunoprecipitation assays. In (**A**), PrP was immunoprecipitated and blotted with anti-Hsp70. In (**B**), Hsp70 was immunoprecipitated and blotted with anti-PrP. **C**. Immunofluorescence. HEK293 cells co-transfected with pEGFP-Hsp70, and various PrP plasmids were stained for 48 h. The individual and merged images of PrP (red), Hsp70 (green), DAPI (blue) were monitored under confocal microscopy. The preparations of the various PrP constructs are indicated on the left. **D**. The cells infected with various PrP plasmids were extracted subcellular proteomes and examined separately by Western blot analysis using PrP-, Hsp70- and Calnexin-specific antibodies. C: cytosol fraction M: membrane/organelle fraction. **E**. Immunoprecipitation assays of cytosol fraction. The cytosol fractions extracted from the cells transfected with various PrP plasmids were to be immunoprecipitated by anti-PrP and blotted with anti-Hsp70.

Hsp70 is required as a structural component of refolding/degradation complexes to correct or clear several misfolded amyloidogenic proteins involved in neurodegenerative diseases. To determine if Hsp70 interacts with PrPs, HEK293 cells without detectable cellular PrPs were co-transfected with pcDNA-PrP-PG5 and pEGFP-Hsp70. Western blots of the cell lysates revealed two Hsp70-specific bands at 70 and 90 kDa, representing the endogenous Hsp70 and recombinant EGFP-Hsp70, respectively. In addition, PrP-specific signals were detected at 25–35 kDa (Figure 1). When PrPs were immunoprecipitated from the cell lysates and subjected to Western blot analysis, two Hsp70 bands were detected (Figure 1A). Similarly, when Hsp70 was immunoprecipitated and subjected to Western blot analysis, PrP specific signals were detected (Figure 1B). Neither Hsp70 nor PrP were detectable in the cell lysates immunoprecipitated with isotype IgG. These results suggest that PrP can form a complex with Hsp70 in cell lysates.

Normally, PrP^C^ is predominantly localized to the cell surface, but some PrP mutants are retained and aggregate in the cytoplasm. To determine if Hsp70 colocalizes with different PrP mutants in the cytoplasm, we used immunofluorescence to examine HEK293 cells transfected with plasmids containing wild-type or various mutant PrP proteins. By confocal microscopy (Figure 1C), most of the wild-type PrPs (PG5-PrP) localized to the cell surface; ER-retained PrP mutants (3AV-PrP and ER-PrP) formed large plaque-like structures with strong signal intensities in the cytoplasm, while Cyto-PrP and PG14-PrP were found in numerous round particles in the cytoplasm. Interestingly, the distribution of Hsp70 in the cytoplasm was obviously influenced by the different PrP constructs. In the cells expressing PG5-PrP, as well as 3AV-PrP and ER-PrP, Hsp70 was dispersed in the cytoplasm and did not significantly colocalize with the 3AV-PrP or ER-PrP-formed deposits. However, in the cells expressing Cyto-PrP or PG14-PrP, Hsp70 colocalized well with the PrP-specific particles in the cytoplasm. The degree of colocalization between Hsp70 and PrP increased in the cells expressing Cyto-PrP. (Figure 1C). To see the distributions of different PrP constructs in various subcelluar fractions, the cytosolic and membrane/organelle protein fractions were separately extracted with ProteoExtract® Subcellular Proteome Extraction Kit (S-PEK). It showed that the majorities of Cyto-PrP and PG14-PrP presented in the cytosolic fraction, whereas PG5-PrP, 3AV-PrP and ER-PrP were mainly detectable in the membrane/organelle protein fraction (Figure 1D). As expected, cellular Hsp70 was observed in the cytosolic fraction. Subsequent immunoprecipitation experiments of the cytosolic fractions of various PrP preparations confirmed the associations between Hsp70 and cytosolic PrP (Cyto-PrP and PG14-PrP), but not in those of other PrP constructs (PG5-, 3AV- and ER-PrP) (Figure 1E).

### Hsp70 selectively reduced the levels of the cytosolic forms of the PrP mutants

**Figure 2:**
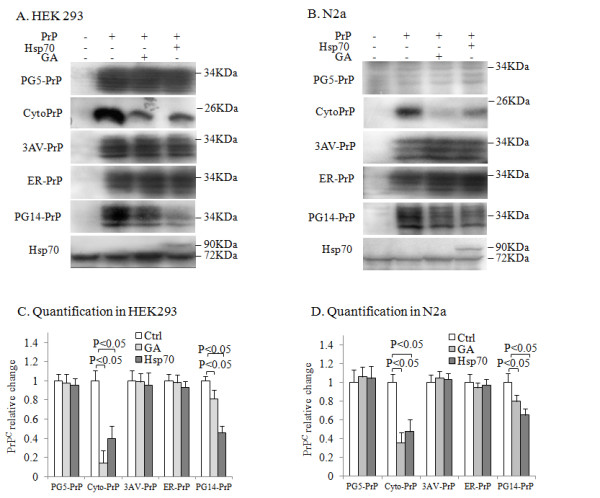
**Hsp70 selectively reduced the levels of the cytosolic forms of PrP mutants. A** and **B**. Western blots. HEK293 (**A**) and N2a cells (**B**) were transiently co-transfected with pEGFP-Hsp70 and various PrP-plasmids. Twenty-four hours after transfection, the cells were treated with or without geldanamycin (1 μM) for an additional 24 h. The cells were harvested and examined separately by Western blot analysis using PrP- or Hsp70-specific antibodies. **C** and **D**. Quantification of the immunoblots. The numerical gray values for PrP were quantified and normalized to β-actin levels in HEK293 cells (**C**) and N2a cells (**D**). All experiments presented in the figures were repeated independently 3 times with consistent results. Each data point represents the mean ± SEM of 3 independent experiments. Samples were considered statistically different from the controls when P < 0.05.

To explore the possible role of Hsp70 in regulating the degradation of various PrPs, we transfected HEK293 and N2a cells with different PrP-expressing plasmids and compared the levels of the PrPs by Western blot analysis. The cells were also treated with geldanamycin (GA), an Hsp90 inhibitor and can indirectly induce Hsp70 expression [15], or co-transfected with the plasmid pEGFP-Hsp70. As expected, the addition of GA increased the levels of endogenous Hsp70 (detected at 70 kDa) while transfection with pEGFP-Hsp70 led to the expression of EGFP-Hsp70 (detected at 90 kDa) in HEK293 and N2a cells (Figure 2A and B). Compared to the controls transfected with PrP-expressing plasmids alone, the levels of PG5-PrP, 3AV-PrP and ER-PrP remained unchanged while the levels of Cyto-PrP and PG14-PrP decreased in HEK293 (Figure 2A) and N2a cells (Figure 2B) treated with GA or transfected with pEGFP-Hsp70. The relative gray values of the PrP signals were quantified and normalized to β-actin levels and revealed significantly lower levels of Cyto-PrP and PG14-PrP in cells treated with GA or transfected with pEGFP-Hsp70 (Figure 2C and D). The result suggests that Hsp70 selectively regulates the metabolism of the cytosolic forms of PrPs but has little effect on the wild-type and ER-retained PrPs.

### Cytosolic PrP degradation mediated by Hsp70 is dependent on ubiquitination and proteasome

**Figure 3:**
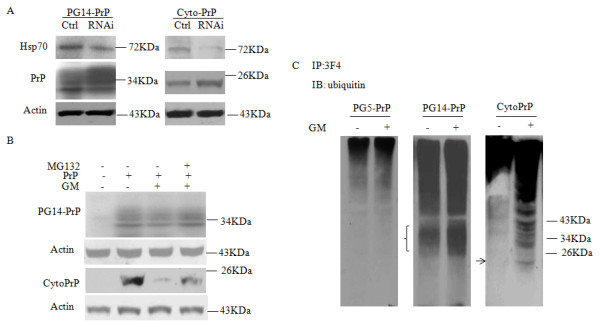
**Ubiquitination and proteasome-mediated degradation. A**. RNA inference. siRNAs for both Hsp70 and negative control RNA were used. HEK293 cells were co-transfected with different siRNA and the plasmid expressing PG14-PrP or Cyto-PrP and harvested 48 h post-transfection. The cell lysates were examined by PrP- and Hsp70-specific Western blots. **B**. Proteasome effect. HEK293 cells were transiently transfected with the plasmid PG14-PrP or Cyto-PrP. 48 h later, 1 μM GA (final concentration) was added into the culture medium and maintained for another 24 h and 30 μM MG132 (final concentration) was added at the last 6 h. The levels of PrP and actin in the cell lysates were evaluated by individual Western blots. **C**. Ubiquitination. HA-ubiquitin and various PrPs were transiently expressed in HEK293 cells in the presence or absence of 1 μM GA. Cells lysates were employed into immunoprecipitation captured with anti-PrP antibody. Ubiquitinated proteins were evaluated by ubiquitin specific Western blots.

To further confirm the role of Hsp70 related to the degradations of cytosolic PrPs, a pair of Hsp70 specific RNAi sequences was synthesized according to the published literature [16]. HEK293 cells were co-transfected with cytosolic PrP constructs (Cyto-PrP or PG14-PrP) and Hsp70 specific RNAi or a commercial negative control RNAi which was assumed to be unable to interfere in the expression of cellular protein. In the cells expressing Hsp70 specific RNAi, the cellular endogenous Hsp70 protein was obviously reduced (Figure 3A), reaching to approximately 60% compared with the cells receiving negative control RNAi. Accompanying with the reduction of Hsp70, both Cyto-PrP and PG14-PrP in the cells expressing Hsp70 RNAi were remarkably increased, compared to those with negative control RNAi (Figure 3A). It indicates that the specific knockdown of the cellular endogenous Hsp70 efficiently block the degradations of cytosolic forms of PrP proteins.

Chaperone proteins-induced misfolded/mislocalized proteins reduction is often a result of ubiquitination-mediated protein degradation. To address whether the Hsp70-mediated degradations of the cytosolic forms of PrP proteins underwent above pathway, the cell lysates of Cyto-PrP and PG14-PrP were subjected to the subsequent tests for evaluating the modifications of ubiquitination and proteasome. Western blots revealed that in the presence of GA, both Cyto-PrP and PG14-PrP were clearly down-regulated, while co-treatment of the cells with MG132, a proteasome inhibitor, was capable of reversing the GA-mediated reductions of Cyto-PrP and PG14-PrP partially (Figure 3B). To see the ubiquitination status of different PrP proteins, a recombinant plasmid expressing HA-tagged ubiquitin (Ub) was transfected into HEK293 cells together with various PrP expressing plasmids. Immunoprecipitation assays using PrP mAb 3F4 as the capturing antibody and ubiquitin pAb as the detecting one showed that in the presence of GA, the signals of the ubiquitinated proteins maintained almost unchanged in the cells expressing wild-type PrP (PG5-PrP) compared to that without GA treatment, while those in the cells expressing Cyto-PrP or PG14-PrP increased markedly, especially in the expected regions of the expressing PrPs (Figure 3C). The data suggest that the derogations of the cytosolic PrPs are mediated by Hsp70 in proteasome after modification by ubiquitination.

### GA selectively reversed apoptosis induced by the cytosolic forms of PrP mutants

**Figure 4:**
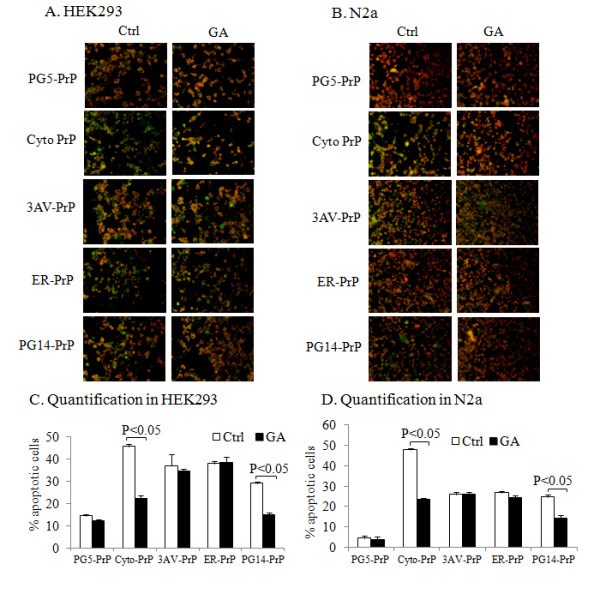
**Geldanamycin selectively reversed the apoptosis induced by the cytosolic forms of PrP mutants. A** and **B**. Assays of mitochondrial dysfunction in the cells expressing various PrP constructs in the presence or absence of geldanamycin. HEK293 cells (**A**) and N2a cells (**B**) expressing various PrP constructs were treated with (GA) or without (Ctrl) geldanamycin, and the mitochondrial transmembrane transition was evaluated with a commercial kit (as described in the Materials and Methods). Apoptotic and living cells are green and red, respectively, by fluorescence microscopy. **C** and **D**. Quantification of the apoptotic cells. The number of total cells in a field of view was counted using ImageJ. The percentage of apoptotic cells was defined as the number of green cells vs. the total number of cells.

To examine the effect of increasing cellular Hsp70 levels on reversing the cytotoxicity of the PrP mutants, the Hsp70 inducer GA was introduced into HEK293 and N2a cells expressing various PrP constructs. The mitochondrial transmembrane potential (MTP) was used to comparatively evaluate apoptosis in cells treated with and without GA. As expected, more green-stained cells were observed in the HEK293 (Figure 4A) and N2a (Figure 4B) cells expressing the mutant than in those expressing wild-type PrPs. There was a significantly higher percentage of apoptotic cells in the mutant PrP-expressing cells. In particular, nearly 50% of the cells expressing Cyto-PrPs were apoptotic (Figure 4C and D). PrP-induced apoptosis was reversed in cells expressing Cyto-PrP and PG14-PrP but not 3AV-PrP and ER-PrP when the cells were treated with 1 μM geldanamycin for 24 hrs (Figure 4). These results suggest that GA reverses PrP-induced apoptosis by enhancing the degradation of cytosolic PrPs.

### Hsp70 proteins are up-regulated in Cyto-PrP-expressing cells and in the brains of scrapie-infected hamsters

**Figure 5:**
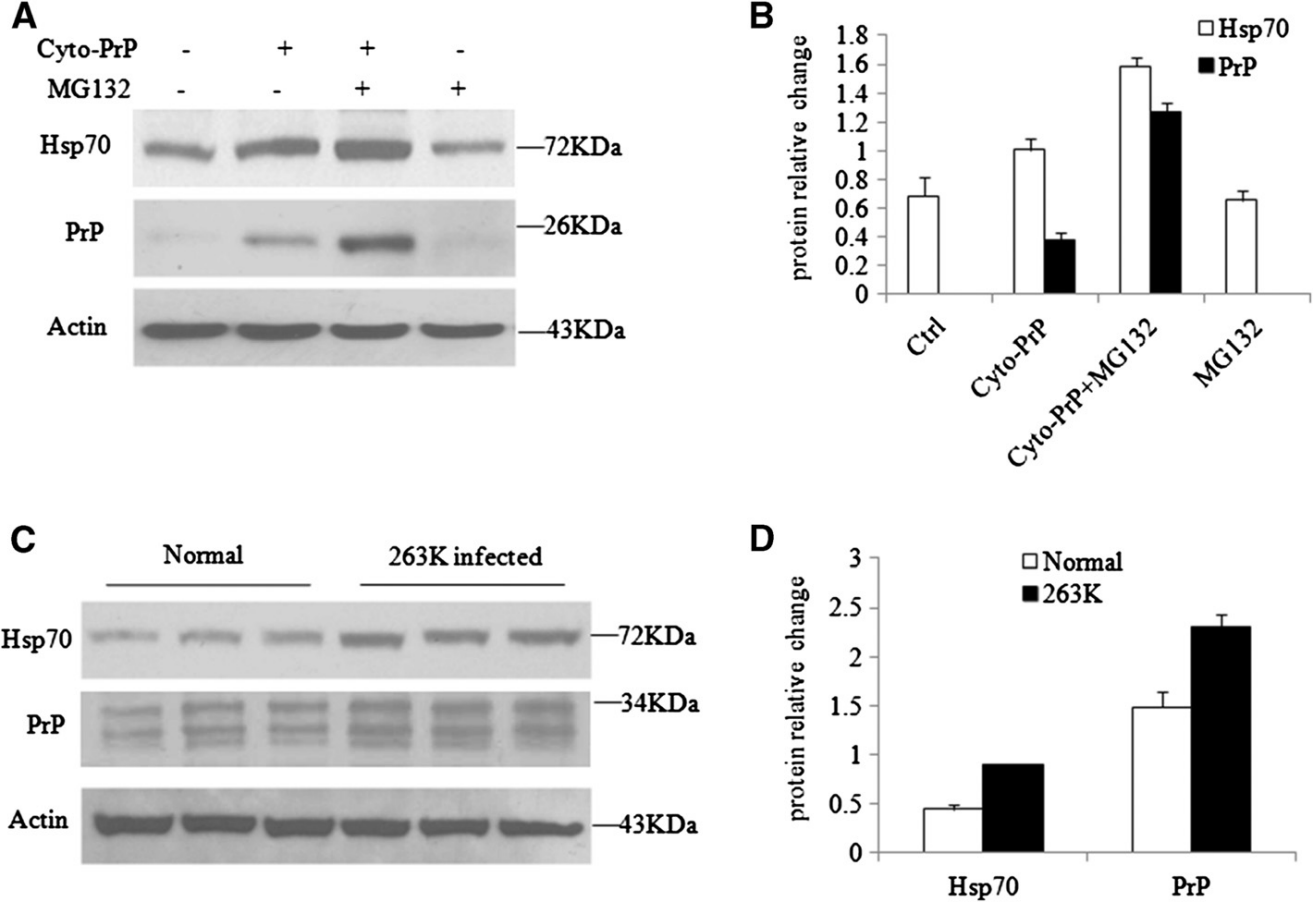
**Hsp70 proteins are up-regulated in HEK293 cells expressing Cyto-PrP and in the brains of scrapie-infected hamsters. A**. Western blots of PrP and Hsp70 in cells expressing Cyto-PrP treated with or without MG132 (10 μM) for 4 h. The cell lysates were separated by 12% SDS-PAGE, transferred onto a nitrocellulose membrane and analyzed by immunoblotting with antibodies against PrP, Hsp70 or actin. **B**. Quantification of the immunoblots. The numerical gray values for PrP or Hsp70 were quantified and normalized to β-actin levels. All experiments presented in the figures were repeated independently 3 times with consistent results. Each data point represents the mean ± SEM of 3 independent experiments. **C**. Western blots of PrP and Hsp70 in the brain tissues of normal and scrapie 263K-infected hamsters. Ten percent of the brain homogenates from each animal was separated by 12% SDS-PAGE and blotted with antibodies against PrP, Hsp70 and actin. **D**. The numerical gray values for PrP or Hsp70 were quantified and normalized to β-actin levels. Each data point represents the mean ± SEM of three animals each group (n = 3).

To determine if the accumulation of PrPs in the cytoplasm affects the expression of endogenous Hsp70, the Cyto-PrP expression plasmid was introduced into HEK293 cells in the presence or absence of the proteasomal inhibitor MG132. We found that the level of Hsp70 detected by Western blots increased in the cells expressing Cyto-PrP (Figure 5A and B). In the presence of MG132, the Hsp70-specific band was much stronger and correlated with the increase in the Cyto-PrP signal. Treating cells with MG132 alone did not change the levels of Hsp70 (Figure 5A and B). This finding indicates that the accumulation of cytosolic PrPs in the cytoplasm will increase the levels of endogenous Hsp70. We next examined the levels of Hsp70 in the brain tissues of scrapie-infected animals. For this study, 10% of the brain homogenates of three scrapie 263K-infected hamsters were used. The gray values detected by Western blotting were quantitated and normalized to β–actin. We found statistically significant higher levels of Hsp70 in the brains of the 263K-infected hamsters compared with the level in normal hamsters (Figure 5C and D).

## Discussion

In the current study, we have demonstrated a molecular interaction between human PrP and Hsp70. Colocalization analyses show that cellular Hsp70 exactly overlaps with the cytosolic forms of PrPs in cells expressing Cyto-PrP and PG14-PrP but not wild-type PG5-PrP or ER-retained PrPs, including 3AV-PrP and ER-PrP. Furthermore, we demonstrate that activation of cellular Hsp70, by overexpression of recombinant Hsp70 or pharmacological treatment with geldanamycin, selectively promoted the degradation of and restored the relevant cytotoxicity of the cytosolic PrPs but not wild-type or ER-retained PrPs in cultured cells. These data indicate that cytosolic PrPs are an authentic substrate of Hsp70.

Hsp70 is a potent molecular chaperone that aids in the folding and refolding of nascent proteins and denatured proteins, respectively. More importantly, when a protein cannot be correctly renatured, Hsp70 can mediate degradation of the protein [17,18]. Misfolded/mislocalized proteins in the cytoplasm are degraded via the ubiquitin-proteasome pathway. It has been repeatedly reported that the overexpression of Hsp70 facilitates the degradation of α-synuclein [18], a risk factor for PD, and eliminates the hyperphosphorylated microtubule-associated protein tau (tau) [17], a primary pathological component of AD. Therefore, activating Hsp70 is one potential strategy and therapeutic target for treating AD and PD. Similar to the abnormal protein deposits found in the brains of patients with AD and PD, the accumulation of PrP^Sc^ in the brain is a predominant neuropathological feature of prion diseases. However, numerous evidence has suggested that PrP^Sc^ is not toxic; however, based on experiments in cultured cells and transgenic mice, abnormal PrPs, e.g., Cyto-PrP [5] or PrPs mutated in the transmembrane region [12] or in the octarepeat region [4], have been proposed to be neurotoxic. These data highlight the importance of clearing continuously produced misfolded PrPs that might be intermediates of PrP^Sc^. Hsp70 may work as an efficient host factor to clear toxic misfolded or mislocalized PrPs in the cytoplasm.

Our results indicate that the accumulation of cytosolic PrPs increases the levels of cellular Hsp70, which may reflect a triggered mechanism for cell defense. Additionally, our study shows that Hsp70 is significantly activated in the brains of scrapie-infected hamsters, which is consistent with what has been observed in the brain tissues of CJD patients and in animal models of TSE [19]. Although PrP^Sc^ is more protease-resistant and cannot be degraded via the Hsp70-mediated ubiquitin-proteasome dependent pathway, several studies have demonstrated that PrP^Sc^ can translocate into the cytosol of prion-infected cells and induce toxicity [20,21]. Up-regulating Hsp70 in cells would help clear out the penetrated PrP^Sc^ or newly formed prion intermediates.

Hsp70 is usually present in the cytoplasm and mediates the efficient modification of misfolded/mislocalized proteins in the cytoplasm. According to this study, this function is highly location specific because Hsp70 did not affect PrP proteins that retain or accumulate in ER. Correlated with the induced degradation of cytosolic PrPs, the viability of cells expressing the cytosolic PrP mutants is markedly improved. Recently, Fernandez-Funez et al. have described that the wild-type PrP can spontaneously convert into an insoluble protease-sensitive isoform in neurons of the transgenic fly brain expressing wild-type hamaster PrP, meanwhile, over-expression of Hsp70 is capable of preventing the accumulation of PrP^Sc^-like conformers and reducing the neurotoxicity, which suggest that Hsp70 may be a therapeutic candidate for prion diseases [22]. However, the complexes of Hsp70 and PrP is detectable in the fraction of membrane microdomains of aged flies’ brains, which seems to be distinct from the data in this study. The exact reason for this discrepancy remains unknown. Hsp70 usually presents in the cytoplasm of mammalian cells [23]. Similarly, Hsp70 is mainly observed in the cytoplasm in the young transgenic PrP flies, but detected also in the microsome of the old flies. Probably due to the wide distribution of transgenic PrP protein in the membrane microdomains in the flies, it eventually forms a stress along with the spontaneous conversion. In that situation, Hsp70 moves across the membranous structures and into organelles. In fact, the stress-caused shift of Hsp70 from cytoplasm to membrane has been already described. In our experimental condition, the PrP constructs located mainly in membrane and organelle (ER) are transiently expressed, which may be too short to induce such “stress”. That might explain why no complex of PrP-Hsp70 is detected in the fraction of membrane/organelles. Additionally, our data also illustrate an increased Hsp70 level in the brains of scrapie strain 263K-infected hamsters. Further assays of co-localization of PrP and Hsp70 in subcellular organelles, as well as in different fractions of brain homogenates will supply more solid conclusion. Moreover, evidence suggests that the conversion of Hsp70’s function in protein-folding to its function as a degradation factor is mediated by the co-chaperone CHIP [24]. A range of cellular agents, e.g., glucocorticoid receptor [25], ErbB2 [26] and tau [27], are known substrates of CHIP. Additionally, DnaJ (Hsp40) is thought to link the Hsp70 chaperone machine to the ubiquitin-proteasome system [28]. The potential contributions of CHIP and Hsp40 to the Hsp70-mediated protection against PrP-neurotoxicity will be a valuable research topic.

## Competing interests

The authors declare that they have no competing interest.

## Authors’ contributions

JZ, KW and YG performed transfection, Western blot, IHC, IP and drafting the manuscript. QS, CT, CC, and CG constructed the various PrP plasmids. BYZ contributed to collection of animal samples. XPD designed the study and critically revised the manuscript. All of the authors read and approved the final version of the manuscript.
